# Seasonal Influenza Vaccine Effectiveness among Children Aged 6 to 59 Months in Southern China

**DOI:** 10.1371/journal.pone.0030424

**Published:** 2012-01-24

**Authors:** Zhicong Yang, Zhiqiang Dong, Chuanxi Fu

**Affiliations:** Guangzhou Center for Disease Control and Prevention, Guangzhou, China; The University of Hong Kong, China

## Abstract

In China the protective effect of seasonal influenza vaccine has only been assessed in controlled clinical trials and proven to be highly effective. However, the post-licensure effectiveness of influenza vaccine has not been examined. In our study all influenza cases from the 19 surveillance sites in Guangzhou were laboratory confirmed during 2009 and 2010. Controls were randomly selected from children aged 6 to 59 months in the Children's Expanded Programmed Immunization Administrative Computerized System. 2529 cases and 4539 controls were finally enrolled. After adjusting for gender, age and area of residence, the vaccine effectiveness of full vaccination was 51.79% and 57.78% in the 2009 and 2010 influenza season, respectively. Partial vaccination provided 39.38% and 35.98% protection to children aged 24 to 59 months in 2009 and 2010, respectively, and no protective effect was observed among younger children. Full vaccination is highly protective and partial vaccination is protective for older children. Influenza vaccination in general should be encouraged, and full vaccination should be particularly encouraged because its protective effect is much stronger than that of partial vaccination.

## Introduction

Influenza causes more morbidity than any other vaccine-preventable illness [1] and leads to an estimated 0.7 to 0.9 hospitalizations, 50 to 95 outpatients visits, 6 to 27 emergency department visits, and 30 to 90 courses of antibiotics each year per 1000 children aged less than 5 years [2], [3], [4]. Rates of influenza-related hospitalizations, acute otitis media, and pneumonia are particularly high in children younger than 2 years of age [2], [4], [5], [6], [7]. Immunization is the major public health measure for the prevention of influenza virus infection. Because the predominant circulating strains of the influenza virus drift over time, influenza vaccination is recommended each year.

**Figure 1:**
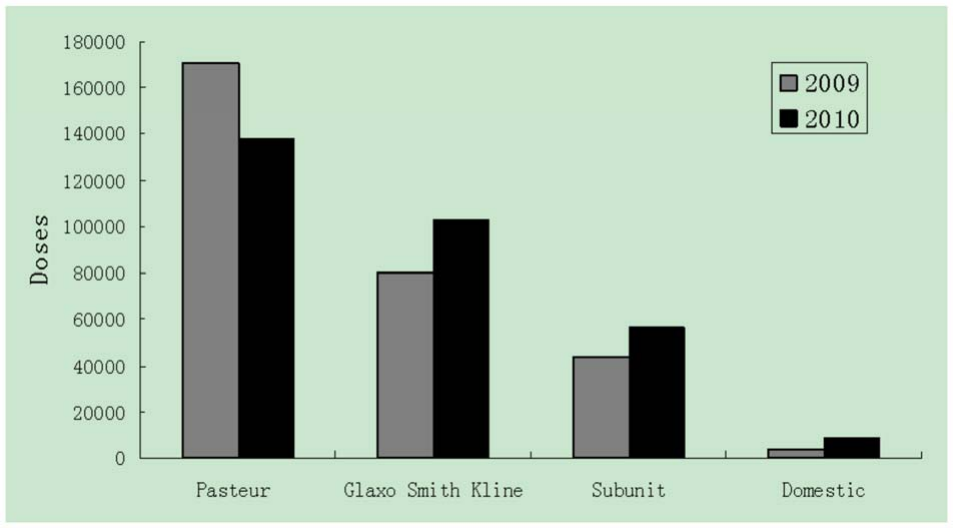
Seasonal influenza vaccine use in Guangzhou during 2009 and 2010.

On the Tropic of Cancer, with over 7.94 million registered inhabitants and a floating population of 4.76 million, Guangzhou is the largest trading city in southern China. Routine influenza virus surveillance data show that in Guangzhou, the influenza season peaks from March to July, and seasonal influenza vaccine usually becomes available before October [8]. Since the Severe Acute Respiratory Syndromes (SARS) epidemic in China in 2003, the population's awareness of influenza vaccination has greatly improved and more influenza vaccinations were administered in 2003 than ever before. According to the Vaccine Management System in Guangzhou Center for Disease Control and Prevention (GZCDC), which dispatches vaccines to all hospitals and health communities in the city, there were 11286, 58570, 84948 and 390138 vaccinations administered from 2000 to 2003, respectively. From 2009 to 2010, there were 603,557 doses of influenza vaccine for children used in Guangzhou, among which 51.2% (308,524 doses) were from Pasteur, 30.3% (182,804 doses) were from GlaxoSmithKline, 16.5%(99,821 doses) were subunit vaccines, and only 2.1% (12,408 doses) were from domestic manufacturers (Figure 1).

In China, the protective effect of seasonal influenza vaccine has only been assessed in controlled clinical trials (seroconversion reports) and has been proven to be highly effective [9], [10], [11]. However, the post-licensure effectiveness of influenza vaccine has not been examined. In this study, we aimed to estimate the vaccine effectiveness (VE) of trivalent inactivated influenza vaccine among children by using a case-control design.

## Materials and Methods

### Study subjects

Nineteen hospitals or health communities located in all 12 districts of Guangzhou City were required to report weekly to GZCDC the total number of consultations and any patients presenting with influenza-like illness (ILI), which was defined as a history of body temperature ≥38°C accompanying with cough or sore throat symptoms (National Influenza Surveillance Plan, 2010, China's Ministry of Health). After the informed consent was signed, participating general practitioners collected data on patients' birth date, gender, and area of residence. We restricted patients in our study to those aged 6 to 59 months. Nose and throat swabs were offered to patients presenting within 3 days of the onset of symptoms. Influenza like illness meeting our case definition reporting to 19 surveillance sites in Guangzhou were subjected to laboratory testing. The presence of influenza virus in the swab samples was detected by by real-time polymerase chain reaction (RT-PCR) and/or isolation of the virus in cell cultures, as described previously [8], [12]. Influenza cases were confirmed by i) RNA of influenza virus, including seasonal H1N1, H3N2 and B; or ii) isolation of the virus.

Controls were randomly selected from children aged 6 to 59 months in the Children's Expanded Programmed Immunization (EPI) Administrative Computerized System as described previously [13]. Controls did not have ILI during the study influenza season, which was confirmed by a phone call by physicians from GZCDC.

### Influenza vaccination status

Influenza vaccination information was retrieved from the computerized system mentioned above, in which the reporting of vaccines administered to children less than 6 years in Guangzhou was required. Influenza vaccination status was classified as fully vaccinated, partially vaccinated or unvaccinated, according to the national guidelines for each season. Children were considered to be fully vaccinated if they received 2 doses of vaccine at least 28 days apart during the influenza season under study, with the second dose given at least 14 days before ILI onset, or at least 1 dose in a previous influenza season and 1 dose in the season under study, administered at least 14 days before ILI onset. Children were considered to be partially vaccinated if they received only 1 of 2 vaccine doses during the current season, and the dose was received at least 14 days before ILI onset, or if 2 doses were received during the current season with the second dose administered within 14 days before ILI onset or less than 28 days after the first dose. Children were considered to be unvaccinated if they were not vaccinated in the study season or if they received the first of 2 doses within 14 days before ILI onset.

### Statistical analysis

We restricted our analysis to patients who presented for medical attention at any of the 19 sentinel surveillance sites and who subsequently had a swab taken for the identification of influenza virus. Data were colleted and aggregated at GZCDC. Exclusion criteria for cases and controls included absence of records in the Children EPI Administrative Computerized System.

Data analysis was conducted using SPSS statistical software (version 13.0, SPSS, Inc., Chicago, IL). We used *χ^2^* and *t* test analyses to compare the characteristics of each group. VE was calculated as one minus the adjusted matched odds ratio (OR)×100%, where the OR was the odds of confirmed influenza among the vaccinated compared with the odds of influenza among the unvaccinated. For all analyses, *P* values not more than 0.05 were regarded as significant.

The protocol of this study was approved by the ethics committee of GZCDC and written informed consent was obtained from the guardians of all children recruited to undergo a swab test.

## Results

Based on the Children's EPI Administrative Computerized System, we identified 1,806 cases (81.2% of total 2,225) and 2,400 controls in 2009, yielding 4,206 total subjects in 2009, and 723 cases (86.7% of total 834) and 2139 controls in 2010, yielding 2,862 total subjects in 2010. Demographic information, such as gender, age and area of residence, was similar between cases and controls. Roughly 12% of cases had received full or partial influenza vaccination before their disease onsets (Table 1).

**Table 1:** Seasonal Influenza Vaccine effectiveness in Children Aged 6 to 59 Months in 2009 and 2010, Guangzhou, China.

Vaccination Status	Age 6–23 mo			Age 24–59 mo			Age 6–59 mo		
	Cases	Controls	VE, Estimate(95% CI),%	Cases	Controls	VE, Estimate(95% CI),%	Cases	Controls	VE, Estimate(95% CI),%
2009									
Fully vaccinated	55(7.78)	126(10.50)	61.01(44.06–72.83)	117(10.67)	262(21.83)	62.43(51.70–70.77)	172(9.52)	388(16.17)	51.79(41.31–60.40)
Partially vaccinated	15(2.12)	17(1.42)	8.52(−31.84–36.53)	35(3.18)	86(7.17)	39.38(25.50–50.68)	50(2.77)	103(4.29)	32.43(19.24–43.47)
Unvaccinated	637(99.10)	1057(88.08)	1.00	947(86.17)	852(71.00)	1.00	1584(87.71)	1909(79.54)	1.00
Male	499(70.58)	600(50.00)		758(68.97)	600(50.00)		1257(69.60)	1200(50.00)	
Age (yr)	1.31±0.49	1.00±0.82		3.68±0.89	3.67±0.47		2.75±1.38	2.33±1.49	
Urban area	374(52.90)	600(50.00)		606(55.14)	600(50.00)		980(54.26)	1200(50.00)	
2010									
Fully vaccinated	17(5.66)	163(16.30)	73.37(54.72–84.34)	45(10.64)	201(17.63)	47.57(25.59–63.05)	62(8.58)	364(17.02)	57.78(43.61–68.39)
Partially vaccinated	9(3.00)	45(4.50)	23.86(−10.53–47.55)	17(4.02)	97(8.51)	35.98(16.33–51.01)	26(3.60)	142(6.64)	32.51(16.10–45.72)
Unvaccinated	274(91.33)	837(83.70)	1.00	361(85.34)	842(73.86)	1.00	635(87.83)	1633(76.34)	1.00
Male	226(75.33)	519(51.95)		285(67.40)	570(50)		511(70.68)	1089(50.91)	
Age (yr)	1.29±0.49	1.12±0.79		3.50±0.90	3.67±0.47		2.58±1.33	2.48±1.42	
Urban area	148(49.33)	519(51.95)		193(45.63)	600(52.63)		341(47.16)	1119(52.31)	

VE was adjusted for gender, age and area of residence (urban or rural area).

The overall vaccine effectiveness of full vaccination compared to no vaccination was 46.57% [95% confidence interval (CI), 35.30–55.89] and 56.20% (95% CI, 41.78–67.04) among children in the 2009 and 2010 influenza seasons, respectively. After adjusting for gender, age and area of residence, the VE of full vaccination was 51.79% and 57.78% in the 2009 and 2010 influenza seasons (p<0.001), respectively.

Partial vaccination provided weaker protection than full vaccination. Partial vaccination provided 39.38% and 35.98% protection to children aged 24 to 59 months in 2009 and 2010, respectively, and no protective effect was observed among younger children.

The VE of full vaccination against influenza virus A was 50.91% (95% CI, 39.39–60.23) and 17.28% (95% CI, −26.06 to 45.72) against influenza virus B in 2009. In 2010, the VE against influenza virus A and B was 57.72% (95% CI, 32.01–73.71) and 56.71% (95% CI, 34.16–71.53), respectively. Our viral surveillance data (unpublished) showed that in Guangzhou, the influenza virus constituent ratios for A (H1N1, H3N2) and B subtypes in 2009 were 73.24% and 26.76%, respectively, based on 3,481 swabs. The ratios in 2010 were 42.02% and 57.98%, respectively, based on 2,689 swabs. However, due to a lack of further information, we cannot determine how well the circulating and vaccine strains of influenza matched.

## Discussion

We analyzed vaccination information for 2,529 laboratory-confirmed influenza cases and 4,539 controls from 2009 to 2010. We discovered that the VE for full vaccination versus no vaccination was 51.79% in the 2009 influenza season and 57.78% in the 2010 influenza season (p<0.001). This post-licensing study of VE found full vaccination to be highly protective and partial vaccination to be protective of children aged 24 to 59 months.

The effectiveness of seasonal influenza vaccine over multiple influenza seasons, especially against laboratory-confirmed influenza, in children younger than 5 years of age has not been well studied. Over the ten years that the vaccine has been used in China, VE has not been reported. Our study, which used a large sample size, is the first to determine the VE of seasonal influenza vaccine in China. In China, many controlled clinical studies on influenza vaccine seroconversion have shown that different seasonal influenza vaccines have similar immunogenicity to three sub-types of influenza virus (H1N1, H3N2 and B) [9], [10], [11].

Pre-licensing studies normally evaluate protection under the optimal conditions of clinical trials. However, the real contribution of a vaccine is better estimated by its performance when used in practice. Efficacy figures from clinical trials cannot easily be converted to VE because during routine practice, not all susceptible children will be immunized before exposure or receive a full immunization program. In addition, the population of vaccine recipients in practice typically expands beyond the healthy, highly responsive groups that are usually selected for efficacy trials [13], [14], [15], [16]. Therefore, from a public health perspective, the impact of vaccination on practical outcomes should be analyzed.

Because there is no pre-licensing study available on influenza vaccine efficacy against confirmed influenza cases, we find it difficult to compare our results with those of previous studies. Bian GL et al. employed a face-to-face interview and a self-administered questionnaire to acquire the incidence of ILI among 470 healthy persons aged 0 to 17 years in Ningbo, China, from December 2002 to September 2003, and found that one dose of seasonal influenza vaccine could protect 79.22% of the subjects [17]. However, detailed vaccination information is not available and the reported cases were not laboratory confirmed. Guo RN et al. applied the Computer Assisted Telephone Interviewing System (CATI) and found that the incidence of ILI among Guangzhou residents in 2006 was estimated to be 22.9%, and the influenza vaccination rate was 7.24%[18]. Unfortunately, in their study, the authors did not report the vaccination rate among cases and the healthy population by age and thus failed to determine the VE against ILI in the city.

Our estimated VEs, based on healthy controls with no ARI symptoms, were consistent with expectations of systematic trials [19], but slightly different from reported findings in developed countries [20], [21], because partial vaccination showed weak protection (about 32%) in children aged 24 o 59 months in our study. Older children might have a better immune response to partial vaccination than younger children. There are two studies with findings similar to ours. A matched case-control study to assess the influenza VE from 1999–2000 through 2006–2007 was conducted in Olmsted County, MN, USA, which confirmed strong protection in the fully vaccinated group against laboratory-confirmed, medically attended influenza among children 6 to 59 months of age. The protective trend continued in the partially vaccinated group (OR: 0.27, 95% CI, 0.07–0.97) [22]. Based on a prospective case-control study in general practices and the emergency department in Western Australia, Heath found that the adjusted VE against laboratory-confirmed influenza among children aged 6 to 59 months was 68%(95% CI, 26–86) [23].

There is one report that did not support the influenza vaccine's protective effect. Szilagyi et al. designed a case-cohort study to estimate effectiveness of inactivated influenza vaccine in preventing inpatient and outpatient visits in 3 counties during the 2003–2004 and 2004–2005 influenza seasons. However, based on 165 and 80 inpatient/ED and 74 and 95 outpatient influenza cases during the 2003–2004 and 2004–2005 seasons, the authors could not demonstrate VE in preventing influenza-related inpatient/ED or outpatient visits in children younger than 5 years [20]. We believe this may be because the sample is not representative. Further study is needed during the coming years.

Selection bias in controls may be reduced by using standardized computer methods to enroll healthy children, as opposed to enrolling those with no confirmed viral infection among those with ARI symptoms [23]. In this study, vaccination status was based on electronic immunization records instead of parents' or guardians' recall, making it unlikely to be affected by recall bias. In addition, the dates of disease onset and vaccination were electronically recorded, which eliminated the possibility of including cases that were out of temporal order with regard to the dates of disease onset and vaccination [13].

There are other limitations to our study. As the 2009 pandemic influenza (A) H1N1 caused significant concern in Guangzhou, a large sample size was obtained during the 2009 study period for children with ARI symptoms. As the concerns decreased, fewer cases of ILI were reported to the surveillance sites in 2010, which may have resulted in a sample of cases of ILI that is not representative. We could not obtain the detailed information needed to determine how well the circulating and vaccine strains of influenza matched, and further study is needed.

We found full vaccination to be highly protective and partial vaccination to be protective among older children. Influenza vaccination in general should be encouraged, and full vaccination should be particularly encouraged because its protective effect is much stronger than that of partial vaccination.
